# The devastating impact of illegal mining on indigenous health: a focus on malaria in the Brazilian Amazon

**DOI:** 10.17179/excli2023-6046

**Published:** 2023-04-06

**Authors:** Paulo Ricardo Martins-Filho, Nicole Prata Damascena, Analany Pereira Dias Araujo, Melina Calmon Silva, Bianca Marques Santiago, Alexandre Raphael Deitos, Carlos Eduardo Palhares Machado

**Affiliations:** 1 Federal University of Sergipe, Sergipe, Brazil; 2 National Center for the Dissemination of Forensic Sciences, Brazilian Federal Police, Distrito Federal, Brazil; 3 National Institute of Criminalistics, Brazilian Federal Police, Distrito Federal, Brazil; 4 Center for Forensic Medicine and Dentistry, Institute of Science Police of Paraiba, Paraiba, Brazil; 5 Federal University of Paraiba, Paraiba, Brazil

## ⁯

The Yanomami Indigenous people in Brazil are experiencing a severe humanitarian crisis, mainly due to illegal gold mining, unauthorized logging and land grabbing, and a lack of access to healthcare and basic preventive measures. This has led to several health issues, including infectious diseases, intestinal parasitosis, malnutrition, mercury exposure, sexual exploitation in exchange for food and natural resources, displacement from their lands, and increased mortality. The Yanomami are also vulnerable to COVID-19 due to the decline in indigenous health funding and limited access to primary health facilities and hospitals (Santos et al., 2021[9]). 

Historically, mercury has been used to amalgamate gold particles in the Brazilian Amazon (Malm 1998[4]), allowing for their easy separation from the ore. However, mercury-based gold mining releases significant amounts of this toxic metal into the environment, where it is naturally converted to methylmercury by microorganisms, resulting in widespread contamination of fish and animals used as food sources and the region's inhabitants (Castilhos et al., 2015[1]). Illegal mining and deforestation also increase the incidence of malaria and other diseases among Indigenous people in the Amazon region (Ellwanger et al., 2020[3]). Mining activity creates a favorable environment for the breeding and spread of Anopheles mosquito species, as the dredging of ravines generates pools of water that serve as artificial breeding sites. Additionally, illegal mining can result in the migration of people to mining areas, leading to an increased population in remote areas of the Amazon region and facilitating the spread of malaria (Murta et al., 2021[7]).

This ecological study aimed to investigate the occurrence of malaria in Indigenous populations living in the Brazilian Legal Amazonia, which includes the states of Acre, Amazonas, Amapá, Maranhão, Mato Grosso, Pará, Rondônia, Roraima, and Tocantins. Indigenous lands occupy approximately 115 million hectares (23 %) of the Amazonian territory, with the Yanomami people owning 10 million hectares and a population of over 27,000 Indigenous people (https://terrasindigenas.org.br/) (Figure 1, supplementary information). Data were extracted from the Epidemiological Surveillance Information System for Malaria (SIVEP-Malaria), an online information system that stores records of all malaria notification forms in Brazil, for the period from 2008 to 2022. The collected data were de-identified. Three analyses were performed: first, we compared the number of cases aggregated over three five-year periods (2008-2012; 2013-2017; and 2018-2022); second, we calculated the annual incidence rate of the disease per 1,000 Indigenous people using population estimates provided by various official sources (https://pib.socioambiental.org/pt/Quadro_Geral_dos_Povos); and third, we utilized the Mann-Kendall test to determine significant temporal trends in malaria cases in mining areas in the Amazon region. The data were imported into the R software for data cleaning, manipulation, and visualization. P-values less than 0.05 were considered statistically significant.

Epidemiological data on the incidence of malaria and illegal mining in the Brazilian Legal Amazonia suggest a catastrophic scenario. Between 2008 and 2012, there were 117,781 cases of malaria in Indigenous lands. Of these, 23,610 cases (20 %) occurred in Yanomami territory. From 2013 to 2017, the number of cases decreased to 93,314, although there was a slight increase among the Yanomami, with 23,981cases reported (25.7 %). In the most recent period, from 2018 to 2022, there was a notable increase in the incidence of malaria cases in Indigenous territories. A total of 173,386 cases have been reported, with a staggering 81,516 of those cases (47 %) affecting the Yanomami population. 

Although the incidence rates of the disease have remained below 100 cases per 1,000 Indigenous people in the last 15 years, there has been a significant increase in the incidence of malaria in Yanomami territory in recent years, with estimates exceeding 500 cases per 1,000 since 2019. These data demonstrate an increased disease burden in Indigenous territories in recent years, particularly affecting the Yanomami population (Figure 2A; supplementary information). Finally, the Mann-Kendall test showed an increasing trend in the percentage variation of malaria cases (compared to the previous year) in mining areas in the Amazon region since 2014 (p = 0.021), with the highest growth rates occurring in 2019 and 2020 (Figure 2B; supplementary information).

More than 99 % of malaria cases in Brazil occur in the Amazon region, and the disease remains a major public health problem. In recent years, there has been significant increase in malaria cases and hospitalizations (de Aguiar Barros et al., 2022[2]), particularly among younger Indigenous men (Meireles et al., 2020[6]). A recent study investigating the changes in the epidemiological profile of malaria in Indigenous areas, including Yanomami lands, found that Indigenous children have a higher incidence of the disease, with over 40 % of cases in those aged 0 to 9 years old (Wetzler et al., 2022[10]). Moreover, evidence shows that at least 70 % of malaria infections among the Yanomami are submicroscopic and asymptomatic, contributing to the maintenance of malaria transmission (Robortella et al., 2020[8]). In addition to Indigenous populations, the number of malaria cases has also increased among miners (Wetzler et al., 2022[10]). It was demonstrated that inadequate knowledge of gold miners about malaria transmission and prevention strategies can create ideal conditions for the disease's persistence (Murta et al., 2021[7]). To address these challenges, a multifaceted approach is necessary, including improved health education and access to accurate diagnosis and treatment. 

Several factors may explain the increase in malaria cases in the Amazon region, including limited access to preventive measures, environmental degradation from intensified economic activities such as mining and logging, and inadequate healthcare services in remote areas that hinder the diagnosis and treatment of the disease. In the North region, illegal mining increased by 1271 % between 1985 and 2020, particularly in the Yanomami, Munduruku, and Kayapó reserves (Mataveli et al., 2022[5]). The underreporting of malaria cases in mining areas is also an issue with serious implications for public health, hindering the allocation of resources needed to prevent and control the spread of the disease. Therefore, better reporting systems and access to healthcare services are necessary, as well as the implementation of molecular methods for identifying malaria infections, which has been characterized by low-density parasitemia and the circulation of multiple species of malaria parasites (Robortella et al., 2020[8]).

In summary, addressing the challenges posed by malaria in the Amazon region will require a comprehensive approach. Improving health education, diagnostic methods, and access to treatment are essential, as well as disease reporting systems in more remote areas. Environmental protection measures, especially against illegal mining, will also be critical to preventing the spread of the disease.

## Declaration

### Conflict of interest

The authors declare no conflict of interest.

### Acknowledgments

This study was conducted under the auspices of the ANP/CNPq Forensic Anthropology and Identification of Persons Research Group.

## Supplementary Material

Supplementary information
