# Supplementary material for: The devastating impact of illegal mining on indigenous health: a focus on malaria in the Brazilian Amazon
Source: EXCLI J. 2023 Apr 6;22:400–2. doi: 10.17179/excli2023-6046 (PMC10279957; doi:10.17179/excli2023-6046)
Supplement: Supplementary information [file EXCLI-22-400-s-001.pdf]

EXCLI Journal 2023;22:400-402 – ISSN 1611-2156
Received: March 24, 2023, accepted: April 05, 2023, published: April 06, 2023

Supplementary information to:

Letter to the editor:

THE DEVASTATING IMPACT OF ILLEGAL MINING ON INDIGENOUS HEALTH: A FOCUS ON MALARIA IN THE BRAZILIAN AMAZON

Paulo Ricardo Martins-Filho[1*], Nicole Prata Damascena[1], Analany Pereira Dias Araujo[2,3], Melina Calmon Silva[2], Bianca Marques Santiago[4,5], Alexandre Raphael Deitos[2,3], Carlos Eduardo Palhares Machado[2,3]

[1] Federal University of Sergipe, Sergipe, Brazil
[2] National Center for the Dissemination of Forensic Sciences, Brazilian Federal Police, Distrito Federal, Brazil
[3] National Institute of Criminalistics, Brazilian Federal Police, Distrito Federal, Brazil
[4] Center for Forensic Medicine and Dentistry, Institute of Science Police of Paraiba, Paraiba, Brazil
[5] Federal University of Paraiba, Paraiba, Brazil

* **Corresponding author:** Prof. Paulo Ricardo Martins-Filho. Universidade Federal de Sergipe, Hospital Universitário, Laboratório de Patologia Investigativa.
Rua Cláudio Batista, s/n. Sanatório. Aracaju, Sergipe, Brasil. CEP: 49060-100.
E-mail: prmartinsfh@gmail.com

https://dx.doi.org/10.17179/excli2023-6046

This is an Open Access article distributed under the terms of the Creative Commons Attribution License (http://creativecommons.org/licenses/by/4.0/).

EXCLI Journal 2023;22:400-402 – ISSN 1611-2156
Received: March 24, 2023, accepted: April 05, 2023, published: April 06, 2023

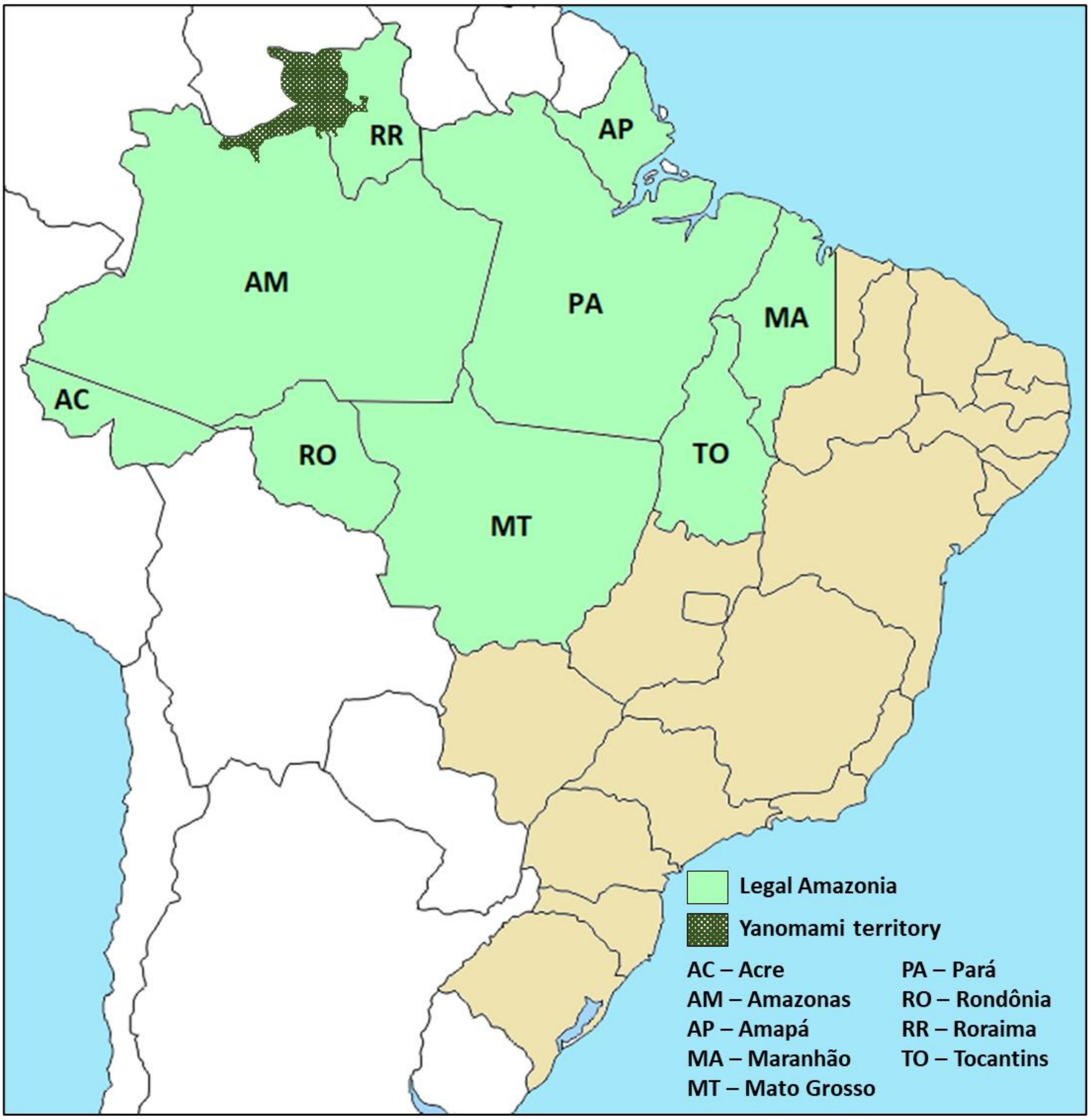

Supplementary Figure 1: Map of the Brazilian Legal Amazon highlighting the Yanomami territory

EXCLI Journal 2023;22:400-402 – ISSN 1611-2156
Received: March 24, 2023, accepted: April 05, 2023, published: April 06, 2023

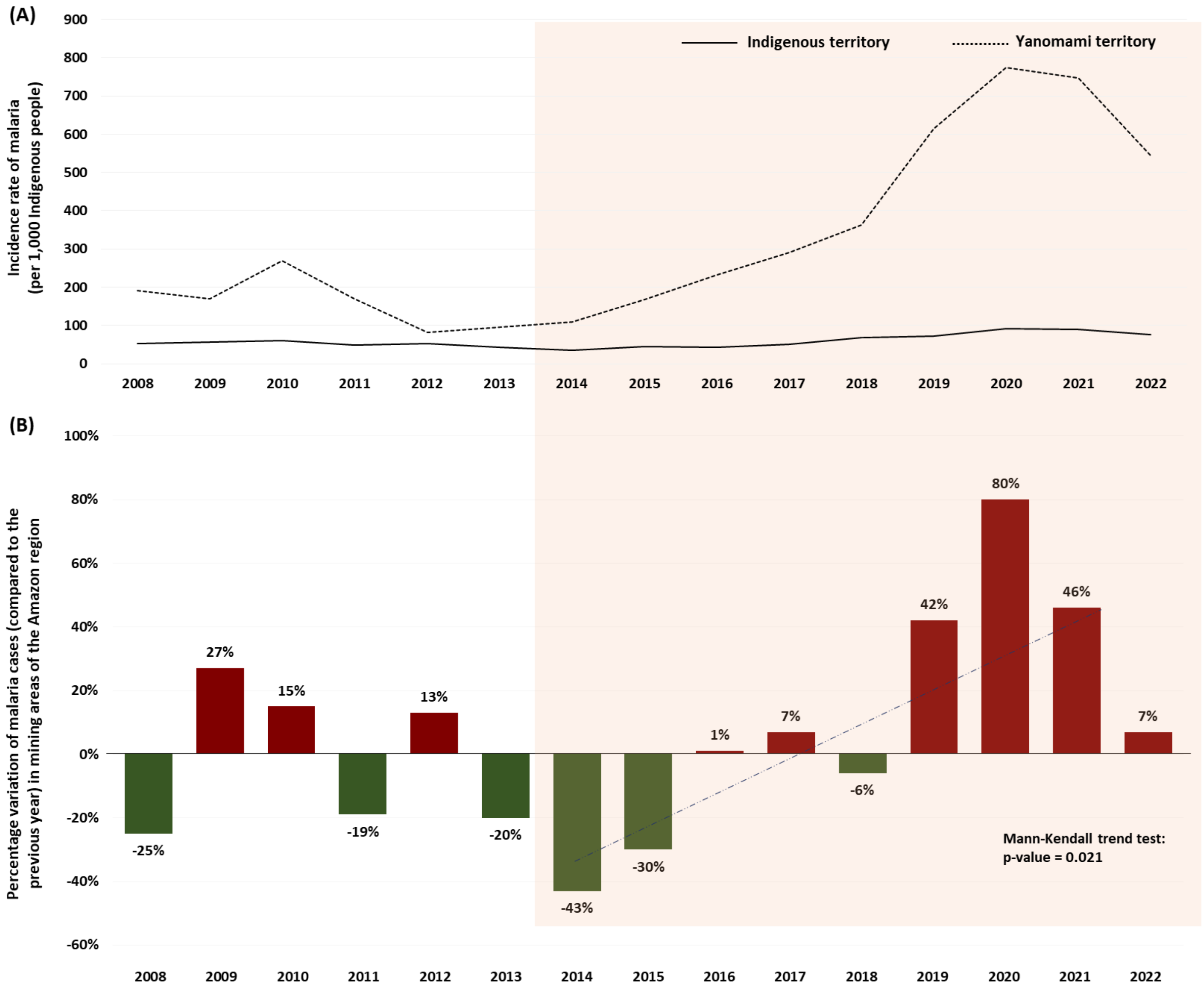

Supplementary Figure 2: Incidence rate of malaria per 1,000 Indigenous people in the Brazilian Legal Amazon (**A**) and percentage variation of malaria cases (compared to the previous year) in mining areas from 2008 to 2022 (**B**).
